# Consumer and staff perspectives of the implementation frequency and value of recovery and wellbeing oriented practices

**DOI:** 10.1186/s13033-018-0244-9

**Published:** 2018-10-20

**Authors:** Keren E. Wolstencroft, Frank P. Deane, Cara L. Jones, Adam Zimmermann, Merrilee Cox

**Affiliations:** 1 Neami National, Wollongong, NSW Australia; 2 Illawarra Institute for Mental Health, School of Psychology, University of Wollongong, Wollongong, NSW Australia; 3 School of Psychology, University of Wollongong, Wollongong, NSW Australia; 4 KPMG Australia Services Pty Ltd., Melbourne, Australia; 5 Mental Health Association of Central Australia, Alice Springs, NT Australia

**Keywords:** Recovery, Wellbeing, Mental health, Service, Practice, Implementation, Translation, Coaching, Training

## Abstract

**Background:**

Despite advances in our understanding of what mental health systems and services can do to enhance recovery and wellbeing outcomes for people seeking support, there is limited evidence demonstrating that this body of work has translated successfully into mental health service practice. The Collaborative Recovery Model (CRM) is a practice framework that has been designed to support application of recovery and wellbeing oriented principles and practices within mental health service delivery. The aims of this study were to assess consumer and staff perceptions of implementation frequency during service engagement and the value of this approach for assisting recovery within a setting where the CRM approach had been adopted.

**Methods:**

The setting was a large Australian community managed mental health organisation. The study involved a cross-sectional analysis of consumer (*n* = 116) and staff practitioner (*n* = 62) perspectives. A series of paired sample t-tests assessed for differences between consumer and staff perceptions of the: (i) importance of key practice elements for assisting recovery, and the (ii) frequency that key practice elements are utilised during engagement sessions. Spearman’s *r* correlational analysis explored associations between importance, frequency and helpfulness of sessions.

**Results:**

Key practice elements of the model were applied during service interactions at a high level and perceived by the majority of consumers and staff participants as being important or very important for assisting recovery. Significant moderate correlations were found between the extent that practice elements were valued and the level at which they were applied. Higher levels of implementation of CRM practices were associated with higher ratings of perceived session helpfulness. The strongest association was between ‘encouragement to set tasks to complete between support visits’ and perceived helpfulness.

**Conclusions:**

Consumer and staff responses revealed that the key practice elements of the CRM were frequently implemented during service engagement interactions and were seen as valuable for assisting recovery. The level of agreement between raters suggests firstly, that the key practice elements were apparent and able to be rated as occurring, and secondly that the CRM approach is seen as responsive to consumer needs. The results have implications for translating recovery and wellbeing oriented knowledge into mental health service practice.

**Electronic supplementary material:**

The online version of this article (10.1186/s13033-018-0244-9) contains supplementary material, which is available to authorized users.

## Background

The activism, advocacy, and narrative based literature generated by people with a lived experience of mental illness and recovery has driven a transformational shift in how mental health and recovery is understood [1–4]. What has emerged is a wealth of valuable knowledge and guidance for what mental health systems and services can do to enhance recovery and wellbeing outcomes for people seeking support [5–10]. However, there is limited research evidence demonstrating that this substantial body of work has, and can be, translated successfully into mental health service practice [11].

Threaded throughout recovery and wellbeing oriented international research and policy literature are two compelling themes fundamental to mental health service practice transformation. Firstly, there is persuasive evidence and argument for a *shift in attention.* That is, mental health services need to broaden their scope of attention beyond illness and symptom alleviation, towards that of enhancing health and wellbeing [3, 12–17]. This requires engagement with complexity over reductionism [18], and deliberate attention to what might be possible for a person seeking support in terms of all that pertains to living well [19]. Secondly, there is well established evidence demonstrating the need for a *shift in engagement style* beyond traditional approaches and positional roles where a person accessing mental health services is seen as a passive recipient of clinical expertise [18, 20, 21]. This shift calls for practitioners to interact in a manner that is person centred and purposefully relational whilst utilising skills and therapeutic processes that are most likely to facilitate hope, self-empowerment and growth [22–26]. Coaching style practices and principles of co-design have emerged as potential facilitators to support these shifts [27–29].

The strength of this knowledge is evident in the health policy missions and standards for mental health care provision in a growing number of countries [30]. However, translating recovery-oriented policy principles and mandates successfully into mental health service systems and practice has proven to be a persistent challenge [31]. Whilst some of the challenges may be typical to implementing evidence based practice in human services in general [32], others are directly related to the nature of the shifts required by recovery and wellbeing oriented approaches [23]. For example, despite extensive efforts to define and translate a broader understanding of recovery into practice, it remains conceptually obscured and operationally entangled within the biomedical paradigm [12, 19, 33–37]. Leaders in the field point out that the shifts that are fundamental to recovery and wellbeing approaches require a ‘whole of service’ and systems orientation [8, 10, 38].

The development of conceptual frameworks and practice models for services and practitioner staff to draw on represent a potentially valuable step in diminishing this knowledge to practice gap [31]. Training supplemented by ongoing learning opportunities such as coaching and supervision have been found to enhance the uptake of evidence based practice approaches [39, 40]. Furthermore, implementation literature in the mental health field also indicates that the degree to which a practice innovation is, (i) championed at the leadership level, (ii) aligned with the values and service operations of an organisation, and (iii) perceived as valuable and relevant by staff and consumers; will influence its uptake and use [41–43].

Aside from well-designed learning and practice development opportunities, of critical importance to practitioner uptake is whether or not an approach is seen as responsive to consumer needs, strengths, personal characteristics and sociocultural context [44]. Positive attitudes towards a practice approach not only increase the likelihood that a consumer will engage with the approach, but also that practitioners will facilitate their use [45, 46]. At present little is known about the link between the value of recovery and wellbeing approaches and consumer perceptions of mental health care [25]. Thus exploring the connection between the value of an approach and its application is important to understanding the barriers and facilitators to successful implementation.

The Collaborative Recovery Model (CRM) is a person centred and strengths based coaching model [23]. The model translates recovery and wellbeing research and policy literature into overarching principles and key processes for mental health services and practitioner staff to implement [47]. The knowledge content and therapeutic structure are informed by lived experience recovery narrative and evidence based practice and positive psychological theories related to change, hope, growth and empowerment processes [4, 7, 48]. The principles and key practice elements are enacted within a coaching-style relationship wherein consumers engage in reflective life visioning and strategic planning processes that are founded on their own aspirations of recovery, wellbeing and/or a good life [23, 49]. The model was piloted and refined by university researchers [7, 42, 50] as part of a National Mental Health and Medical Research Council Strategic Partnership Grant in Mental Health and then adopted by a large Australian community managed mental health organisation as a framework to guide service practice in 2009.

Despite extensive attention to identifying the key elements for practice and the development of models, frameworks and guidelines aimed at improving mental health service practice, there is as yet limited literature demonstrating that services and practitioner staff are enacting these in everyday practice [11, 26, 31]. If mental health services are to give primacy to improving wellbeing outcomes for the people they provide support to, then new understandings of what supports people to move from illness to health requires evidence for their translatability to real world service settings and value to the people who use such services [14, 17, 51–53].

This paper presents findings from a large study aimed at exploring implementation fidelity to the Collaborative Recovery Model within an Australian community managed mental health organisation, Neami National. Implementation fidelity refers to the extent to which a practice model is being delivered as intended by its designers [54]. Fidelity assessments can measure the presence and strength of an approach as it is used in daily practice, and are fundamental to knowing whether an approach has contributed to intended outcomes [55].

The full study integrated multiple units of analysis—(1) the perceptions of consumers and practitioner staff regarding the *importance* of the model’s key practice elements for assisting recovery and the *frequency* by which these are applied, (2) a file audit of consumer support plans to examine whether self-reports of practice application were consistent with documentary evidence, and, (3) the perceptions of staff across management and non-direct service delivery roles regarding policy and process transformations that have arisen as a result of an organisation wide systems approach to implementation of the model.

This paper focuses attention on the first unit of analysis and seeks to answer four questions:

1. Are the key practice elements within the CRM considered important in assisting recovery?
2. To what extent are the key practice elements of the CRM applied during support engagement activities?
3. Is there any relationship between how the key practice elements of the CRM are valued and the frequency by which they are applied?
4. Is there any relationship between the frequency in which the key practice elements are implemented and consumer perceptions of helpfulness of support engagement sessions?

## Methods

### Design

The method was designed to assess the frequency by which the Collaborative Recovery Model was implemented, and its value for assisting recovery, from the viewpoints of consumers and staff receiving and delivering support framed by this approach. A cross-sectional survey was conducted that assessed the parallel between consumer and practitioner staff perceptions. The study was reviewed and approved by University Human Research Ethics. The procedure outlines the protocols implemented with respect to ethical considerations relevant to this study.

### Procedure

The study was undertaken within an Australian community managed mental health organisation. The organisation is one of the many community managed organisations (also referred to as non-government organisations or NGO’s) in Australia that provide support services to people living with and/or experiencing mental illness and distress. The Australian mental health service sector includes those delivered by government i.e. Public Health System; those delivered privately e.g. private psychiatrist; and those that are delivered by community managed organisations. Community-managed organisations are typically not for profit and deliver supports from a psychosocial framework of care. In Australia the majority of mental health service consumers access mental health support through the public and private sectors with approximately 3.1% of psychosocial mental health services expenditure spent in the NGO sector [56].

At the time of the study, the organisation provided support to 3512 consumers and employed 607 staff from 36 service site locations ranging from inner-city and suburbs to regional and remote areas across five states of Australia. Seven sites were excluded from the study due to service parameters that required non-standard use of the CRM. This included short stay residential services and homelessness outreach services. A proportional random selection process was undertaken to select 12 sites from the remaining 29. Service site participation distribution across states included; Western Australia [1], Queensland [1], South Australia [2], New South Wales [4] and Victoria [4]. Researchers with a lived experience of mental illness and recovery (otherwise named as consumer researchers) were employed in each state to undertake recruitment and data collection activities.

Consumer researchers were employed and renumerated in line with industry based rates commensurate to researcher responsibilities. The consumer researchers had no prior experience in receiving services or being employed by the organisation involved in this study. This procedure was adopted to create an arms-length between service delivery practices and research activities to minimise the potential for bias, maximise confidentiality and safety for participants and minimise potential coercion and/or perceptions of coercion. Furthermore, consumer participants have been found to respond more truthfully when research activities are undertaken with a consumer researcher [57, 58].

Names of ‘active’ consumers from the 12 sites were extracted from the organisational database, Carelink+, and a randomisation procedure was applied to sort consumer names in ascending order for invitation to participate. Active consumers are those who are currently receiving support as opposed to those who have completed their service engagement or on a wait list to receive support. The extraction of consumer names and the randomisation procedure was undertaken by an employee from the study setting holding custodial responsibility for the collection, management, storage and transmission of all administrative data that is collected for service operations across the organisation. In this role the employee has responsibility to implement appropriate physical, technical and ethical safeguards to protect the confidentiality and integrity of organisational data. The funder and the researchers involved this project had no influence over the list creations and randomisation procedure other than providing the parameters related to length of time in service (minimum 3 months) and requirement for participants to be ‘active consumers’ as opposed to no longer receiving services or on a waiting list. Sorted lists were provided in Excel spreadsheets directly to consumer researchers and included consumer names, contact details, ID codes for later use and names of staff practitioners who were the key workers for each consumer. Consumer researchers requested site managers advise them about any consumers currently in hospital. Those names were also omitted from the lists.

Consumer researchers started at the top of the list to contact consumers by phone and invite participation. They introduced themselves to participants as having a lived experience of mental illness and recovery and being employed as a researcher by the service development department of the organisation. After introducing themselves, they provided verbal information about the study and what participation would involve.

For consumers who indicated interest in knowing more, a meeting time and place was set for the consumer researcher and consumer to discuss the project more fully. Consumers were provided with full information (including written) about the study and what participation would involve, invited to discuss and ask questions, and offered time to consider and talk to significant others (e.g. family) about whether or not they would want to participate. Consumer participants were asked to relate back to the researchers their understanding of the nature of the study and what participation involved prior to providing formal and signed consent. Researchers stopped recruitment once participation numbers reached 10 per site. In total 263 consumers were contacted about the study and 116 (44%) agreed to participate and provided signed consent. One participant withdrew from the study due to a change in their circumstances.

Once a consumer had completed participation in the study, consumer researchers contacted staff members who were identified in the Excel spreadsheet as being the key worker for the participating consumer. After introducing themselves and advising the staff member that a consumer they worked with was involved in the study they provided verbal information about the nature of the study and what participation would involve. If a staff member indicated interest they met with a consumer researcher and were provided with full study and participation information (including written) and offered time to consider their decision. All staff participants were taken through a formal information and consent process involving signed consent to participate.

Staff participants were the key workers holding primary responsibility for the coordination and delivery of support to the consumer participants in this study. Key workers at this organisation may be responsible for a portfolio of between five and fifteen consumers. There was 65 staff identified on the Excel lists as being key workers to the 116 consumers who participated in the study. Two staff had left the organisation at the time of recruitment and one did not consent to participate. In total, 62 out of 63 staff members invited to participate did so. Staff participants completed separate measures for each consumer participant they were key worker for. As such, staff participants often completed measures in reference to more than one consumer participant.

In all communications between researchers and participants the voluntary nature of participation was clearly communicated along with options for withdrawing consent. All participants were reminded that a decision to not participate would have no impact on their relationship with the organisation of this study setting, on the service they receive (consumer participants) or their employment (staff participants). Participants were informed of protocols to protect participant confidentiality including the (1) designation of private rooms or option for alternative location of choice (e.g. private room in local library), (2) use of participant ID no. on all paper based and data-based data with only researcher access to the ID key, (3) all paper-based data stored in locked filing cabinets with only researcher access (4) signed consent forms stored separately to paper-based data responses.

Following information and consent processes, consumer researchers met separately with consumer and staff participants to undertake the completion of measures used in this study. All recruitment, information and consent processes, and data collection activities were undertaken in settings ensuring privacy and comfort for participants. No service delivery staff member had access or knowledge of consumer participant responses. Likewise no consumer participant had access or knowledge of staff member responses. Information about who did not consent to participate was only known to the consumer researchers involved in this study.

### Measures

The Consumer Evaluation of CRM (CEO-CRM) and the Staff Evaluation of CRM (SEO-CRM) are matched self-report questionnaires, developed by university and consumer researchers [45] to assess consumer and staff perspectives on key aspects of support as informed by the Collaborative Recovery Model (CRM). The original version includes 15 items related to seven key practice elements of the model. A consumer review of the measures found high levels of acceptance for use [45]. Reliability of the questionnaire was tested as part of previous study and was found to be acceptable (Cronbach’s alpha α = .78 for ‘importance’ items and α = .80 for ‘frequency’ items) [45].

In preparation for this study, we consulted with three consumers, three staff and five consumer researchers to review the measures. Some small adjustments were made to the wording and sequence of the statements including a change in the Likert rating scale for ‘importance’ ratings. The option for ‘somewhat important’ was removed because it was considered too similar to ‘moderately important’. After additional consultation with the university researchers and developers of the CRM, two additional practice elements were also added. These related to values clarification and strengths identification that were not in the original version but considered key practice elements to the CRM. Supplementary files present the CEO-CRM and SEO-CRM measures (see Additional files 1, 2). Table 3 presents the nine key practice elements that form the basis of the modified CEO-CRM and SEO-CRM measures with items 5 and 7 being the additions. The modified CEO-CRM asks consumer participants to firstly provide responses in relation to how personally *important* they perceive nine key CRM practice elements as assisting their recovery according to a 4-point Likert scale; 0 = not important, 1 = moderately important, 2 = important, 3 = very important. The measure then asks consumer participants to rate the *frequency* in which the same nine key practice elements are applied during engagement sessions according to a 5-point Likert scale; 0 = never, 1 = occasionally, 2 = sometimes, 3 = usually, 4 = always. An engagement session is a time designated for a consumer and staff member to meet face to face for the consumer to receive mental health and wellbeing support. Table 1 presents the importance and frequency items. Following completion of *importance* and *frequency* ratings consumer participants are asked to rate how *helpful* the sessions with their key worker had been towards assisting their personal recovery process over the past 3 months according to a 4-point Likert scale; 0 = not at all helpful, 1 = moderately helpful, 2 = helpful, 3 = very helpful.

**Table 1:** Demographic profile of 116 consumer participants

Characteristics	*M*	*SD*	Range
Age	42.9 years	11 years	20–69 years

Characteristics	Frequency	Percentage (%)
Gender		
Female	52	45
Male	64	55
Primary diagnosis		
Schizophrenia	48	41
Depression	23	20
Bipolar	16	14
Schizo-affective	13	11
Anxiety	3	3
Other	13	11
Length of time receiving support		
Less than 1 year	12	10
1–2 years	53	46
2–3 years	30	26
3–4 years	12	10
5+ years	8	7
Missing	1	1
Language other than english		
Yes	14	12
No	89	77
Missing	13	11
Indigenous status		
Yes	7	6
No	109	94

As a matched measure the modified SEO-CRM includes the same nine statements as the CEO-CRM and asks staff participants to refer to an individual consumer participant that they are key worker for in their responses. Staff are firstly asked to rate how *important* they perceive the nine key practice elements of the CRM are for the individual consumer according to a 4-point Likert scale; 0 = not important, 1 = moderately important, 2 = important, 3 = very important. Secondly, staff are asked to rate how rate frequently they deliver the nine key practice elements of the CRM during engagement sessions. Upon completion of importance and frequency ratings staff are asked to rate how helpful they perceive the sessions have been towards assisting the consumer participants recovery process over the past 3 months according to a 4-point Likert scale; 0 = not at all helpful, 1 = moderately helpful, 2 = helpful, 3 = very helpful.

### Data analysis

As this component was predominantly descriptive the main requirements were to seek a representative sample of consumers achieved through random selection. An estimate of sample size was based on previous research which identified between group differences on components of the CRM, using the measures used in the current study with a sample size of *n* of 92 [45]. A series of paired sample t-tests tested for differences between consumer and key worker perceptions of the: (i) importance of key CRM practice elements for assisting recovery, and the (ii) frequency that key CRM practice elements are utilised during engagement sessions. For these comparisons a Bonferroni adjusted p-value of .003 was used. Spearman’s *r* correlational analysis was conducted to assess relationships between variables.

## Results

### Demographic profile of participants

Participants of the current study were active consumers (*n* = 116) and staff practitioners (*n* = 62) of an Australian community managed mental health service. ‘Active consumers’ refers to consumers who were receiving support at the time of the study as opposed to those who had completed their service engagement or were on a wait list to receive support. Participant demographics are presented in Tables 1 and 2.

**Table 2:** Demographic profile of 62 staff participants

Characteristics	*M*	*SD*	Range
Age	37 years	10.8 years	20–63 years
Length of time employed at study setting	2.46 years	2.56 years	1 month to 16 years

Characteristics	Frequency	Percentage (%)
Gender		
Female	37	60
Male	25	40
Cultural and linguistic diversity		
Yes	11	18
No	47	76
Missing	4	6
Highest level educational background		
Postgraduate	15	24
Undergraduate	24	39
Diploma	20	32
Secondary school	3	5
Qualification field		
Social work	10	16
Community welfare	15	24
Counselling	9	15
Psychology	9	15
Sociology	4	6
Other^a^	12	19
Missing	3	5

^a^Other qualifications spread over seven fields with two or less participants per field

The 62 staff participants were the practitioner staff providing mental health service supports to the consumer participants in this study. Out of the 62 staff participants, 61 had attended 3 day induction training in CRM, 54 had attended at least one CRM 6 month booster, and 48 had attended at least one CRM annual booster. The CRM training programme is intended to enhance and reinforce practitioner staff knowledge, skills and confidence to utilise the CRM therapeutic approach. Staff also engage in fortnightly coaching sessions which allow them to discuss consumer progress, and their own personal development within a CRM framework [39].

### The importance of key practice elements of the CRM for assisting recovery

Table 3 provides consumer and staff participant mean importance ratings and standard deviation (SD) for each of the key practice elements. A paired t-test revealed that there was no significant difference between the total item means of consumer and staff ratings of importance.

**Table 3:** Means and standard deviations for importance ratings of key CRM practice elements

Key practice elements	Importance				
	Consumer		Staff		
	*M*	*SD*	*M*	*SD*	*n*
1. Encouragement to take charge of own wellbeing and recovery	2.35	.70	2.43	.75	109
2. Involvement in considering choices and decisions about my recovery	2.49	.62	2.66	.58	109
3. Respect shown for right not to have to take advice	2.22	.74	2.62*	.61	108
4. Help with motivation	2.38	.70	2.26	.85	109
5. Help to reflect/clarify what is important to me	2.24	.79	2.42	.70	109
6. An understanding of my range of needs	2.55	.62	2.49	.69	109
7. Help to identify my strengths	2.18	.85	2.38	.74	109
8. Encouragement to set goals that are personally meaningful to me	2.42	.70	2.45	.73	109
9. Encouragement to set tasks to complete between support visits	1.96	.84	1.91	.89	107
Total	20.80	6.67	21.66	6.58	

Importance scale: 0 = not at all important, 1 = moderately Important, 2 = important, 3 = very Important

*n* for each item includes the number of paired responses obtained for each item

Total was mean of available items

* Significant difference Bonferroni adjustment p < .003

Results indicated that the vast majority of consumer participants rated all key practice elements of the CRM as important in terms of assisting their recovery. The percentage of consumer ratings in the important and very important range was 87% with a range of (76–91%) across elements. On average, they were rated by consumers as not important 4% of the time (range across elements 0–7%). Staff participant ratings of importance were similar with the percentage of ratings in the important and very important range also being 87% with a range of (70–96%) across elements.

Paired-sample *t*-test’s using Bonferroni adjusted alpha levels of .003 were conducted to compare consumer and staff perceptions of importance at the level of individual items. Results indicated no significant differences between mean ratings of importance for eight out of the nine items. However ‘respect shown for right not to have to take key worker advice’ was regarded more highly by staff participants than consumer participants *t* (107) = − 4.46, *p *< .001, with a medium effect size, Cohen’s *d *= .59.

### Application frequency of key practice elements of the CRM

Consumer and staff participant mean frequency ratings for each of the key practice elements are presented in Table 4. There were no significant differences between the total item means of consumer and staff ratings of application frequency. Consumer results showed that key CRM practice elements were perceived as being applied during support engagement activities with their key worker either usually or always on average 79% of the time (range 62–91%). On average consumers rated the elements as never being applied 2% of the time (range 1–10%). Staff participants rated themselves as applying the key practice elements either usually or always on average 80% of the time (range 58–99%).

**Table 4:** Means and standard deviations for application frequency ratings of key CRM practice elements

Key practice elements	Application frequency				*n*
	Consumer		Staff		
	*M*	*SD*	*M*	*SD*	
1. Encouragement to take charge of own wellbeing and recovery	3.17	.90	3.33	.87	107
2. Involvement in considering choices and decisions about my recovery	3.35	.89	3.76*	.56	107
3. Respect shown for right not to have to take advice	3.32	1.04	3.80*	.53	108
4. Help with motivation	3.49	.72	2.84*	1.11	106
5. Help to reflect/clarify what is important to me	3.18	.91	3.00	.94	108
6. An understanding of my range of needs	3.24	.84	3.11	.75	108
7. Help to identify my strengths	3.06	1.05	2.96	.86	107
8. Encouragement to set goals that are personally meaningful to me	3.23	.90	3.28	.85	108
9. Encouragement to set tasks to complete between support visits	2.72	1.24	2.69	1.05	108
Total	28.77	8.61	28.73	7.57	

Frequency scale: 0 = never, 1 = occasionally, 2 = sometimes, 3 = always

*n* for each item includes the number of paired responses obtained for each item

Total was mean of available items

*Significant difference Bonferroni adjustment p < .003

Paired-sample *t*-test’s using Bonferroni adjusted alpha levels of .003 were conducted to compare consumer and staff participant ratings of application frequency at the individual item level. Results indicated no significant differences between consumer and staff mean ratings of application frequency for six out of the nine items. However, there was a statistically significant difference for the ratings of application for three elements.

The key practice element ‘help with motivation’ was on average rated as being applied more often by consumers than staff rated themselves as applying it *t* (105) = − 4.94, *p *< .00; with a substantial effect size, Cohen’s *d *= .69. The element ‘involvement in considering choices and decisions about my recovery’ was rated as being applied less often by consumers than staff rated themselves as applying it, *t* (106) = − 4.57, *p *< .00, with a medium effect size, Cohen’s *d *= .58; and ‘respect shown for right not to have to take practitioner advice’ was also rated as being applied less often by consumers, than staff rated themselves as applying it, *t* (107) = − 4.31, *p *< .00, with a medium effect size, Cohen’s *d *= .55.

### Relationship between how key practice elements of the CRM are valued and the frequency by which they are applied

Table 5 presents Spearman’s *r* correlations between consumer and staff ratings of overall importance and frequency. Significant moderate positive correlations were found between consumer importance and consumer application frequency ratings (*r* = .50) and between staff importance and staff frequency ratings (*r *= .49). Small but significant positive correlations were found between staff frequency ratings and consumer importance and frequency ratings (ranges = .20– .25).

**Table 5:** Spearman’s r correlations between consumer and staff participant importance, frequency and helpfulness ratings

	Consumer importance	Consumer frequency	Consumer helpfulness	Staff importance	Staff frequency
Consumer frequency	.51**	–			
Consumer helpfulness	.24**	.39**	–		
Staff importance	.17*	.20*	.05	–	
Staff frequency	20*	.25**	.04	.49**	–
Staff helpfulness	.14	.18*	.09	.21*	.33**

Listwise *n* = 106

*p < .05 1-tailed **p < .01 1-tailed

### Relationships between the frequency that key practice elements are implemented and perceptions of helpfulness of sessions

Ninety-three percent (*n* = 103/111) of consumer participants perceived sessions with their key worker as being helpful or very helpful (*M* = 2.52 out of 3, *SD* = .60) towards assisting their personal recovery, 5% as moderately helpful (*n* = 6/111) and only 2% as not at all helpful (*n* = 2/111). Staff ratings were lower (*M* = 2.0, *SD* = .69) with 76% (*n* = 84/110) perceiving sessions as being helpful or very helpful and 24% (*n* = 26/110) as being moderately helpful.

A series of Spearman rank-order correlations found that perceptions of ‘helpfulness of sessions’ were positively associated with CRM implementation frequency. Consumer helpfulness ratings were correlated with consumer importance and frequency ratings and staff helpfulness ratings were correlated with staff importance and frequency ratings. For consumers, higher implementation frequency of eight of nine key practice elements showed significant (small to moderate) associations with higher perceptions of helpfulness of sessions. The strongest association with helpfulness was for ‘encouragement to set tasks to complete between support visits’. For staff, significant albeit small associations were found between higher implementation of six of the nine key practice elements with higher perceptions of helpfulness. There was a greater proportion of items showing associations between application frequency and helpfulness, than that of importance and helpfulness. Table 6 presents the correlation coefficients between (1) importance of practice elements and helpfulness of sessions, and (2) implementation frequency and helpfulness of sessions for consumers and staff.

**Table 6:** Correlation coefficients between importance and implementation of CRM practice elements with helpfulness of sessions

Key CRM practice elements	Helpfulness of sessions			
	Frequency		Importance	
	Consumer	Staff	Consumer	Staff
1. Encouragement to take charge of own wellbeing and recovery	.20*	.22*	.32**	.16
2. Involvement in considering choices and decisions about recovery	.13	.01	.04	.09
3. Respect shown for right not to have to take advice	.21*	.18	.17	– .03
4. Help with motivation	.32**	.12	.29**	.13
5. Help to reflect/clarify what is important	.30**	.25**	.06	.20*
6. An understanding of range of needs	.28**	.32**	– .02	.10
7. Help to identify strengths	.30**	.24*	.10	.15
8. Encouragement to set goals that are personally meaningful	.30**	.33**	.13	.09
9. Encouragement to set tasks to complete between support visits to achieve goals	.40***	.19*	.24*	.13

Paired *n*’s range from 106 to 110

* p < .05, **p < .01, ***p < .001

## Discussion

### Importance

The Collaborative Recovery Model (CRM) promotes consumer and staff engagement with an individualised and holistic approach to recovery and wellbeing [23]. Results for the first question revealed that the key practice elements of the CRM were valued at a high level by the majority of consumers (87%) and staff (87%) participants for supporting recovery. The results validate the design of the model as being responsive to consumer needs [44] and align with consumer narrative and recovery literature calling for mental health services to move beyond symptom alleviation [4, 12, 15].

At the individual item level, results indicated no significant differences between consumer and staff perceptions of importance for eight out of the nine key practice elements. However, for one item; ‘involvement in considering choices and decisions about recovery’, it was found that although consumer ratings of importance for this were high (M = 2.49 out of 3) staff ratings were higher (M = 2.66 out of 3). Central to the concept of empowerment is a person having the opportunity to voice their ideas, opinions and concerns [24]. This finding may indicate that when consumers experience this element as business as usual, they may not perceive its value at the same level as staff who receive CRM training which specifically highlights the history of consumer disempowerment and the crucial role of consumer involvement in decision making towards recovery and wellbeing.

### Frequency

For the second question, the majority of consumers (79%) and staff (80%) rated the key practice elements as being applied during service interactions, as either usually or always. There was no significant difference between the total item means between consumers and staff. This represents a substantial agreement between consumer and staff perceptions of application and suggests implementation of the model is detectable and able to be rated as occurring. The model’s use of applied positive psychology constructs, such as cultivating a growth mindset and a focus on values and strengths, provides a framework for translation at the organisational, staff and consumer levels [23].

A component of the model that may have contributed to these results is the expanded use of the ‘parallel process’ [59]. Further to formal training and booster sessions staff practitioners engage in regular coaching, wherein they use exactly the same protocols that they are asked to use with consumers, for their own professional development or personal growth [39, 59]. Aside from developing skills and confidence to use the protocols in their work with consumers, staff are exposed to the tensions and rewards inherent to these processes [59]. There is also an implicitly normalising rationale to this process that confirms that people with mental health difficulties are fundamentally the same as anyone else; that is, everyone has strengths, values, needs and aspirations [23]. Research examining the translation of evidence-based practices, has found that providing staff with ongoing coaching in a practice approach promotes implementation [40]. In the current study, it is possible that providing training in the CRM that was supplemented with ongoing ‘parallel process’ coaching may have contributed to the level of implementation [39].

Paired-sample *t*-test’s comparing consumer and staff application frequency ratings at the individual item level revealed no significant difference between consumer and staff perceptions of application frequency for six out of the nine elements. However, there was a disparity between consumer and staff reports for the application of three elements. Firstly, ‘help with motivation’ was on average perceived by consumers as being applied more frequently during engagement interactions than staff rated themselves as applying it. For practitioners implementing the CRM approach, the ability to engage in discussions and activities that enhance and maintain motivation, are supported through ongoing training. The content of training includes theoretical concepts emerging from the recovery literature and align with knowledge from the positive psychology and wellbeing sciences pertaining to hope, change and growth [48]. Practitioner skill to utilise these concepts during engagement interactions is facilitated through development of coaching and motivational interviewing skills [23]. The disparity in findings indicate that the application of ‘help with motivation’ may be experienced differently by consumers than staff. It is possible that staff predominantly perceive their application of this element as tied to the use of coaching and motivational interviewing skills, whereas consumer perceptions may be formed by a more global sense of ‘help with motivation’ that arises from all aspects of the interaction. This interpretation aligns with literature and evidence for the value of the therapeutic alliance to consumer engagement and satisfaction with a practice approach [18, 25, 60].

The key practice elements ‘involvement in considering choices and decisions about my recovery’ and ‘respect shown for right not to have to take staff advice’ were rated on average by consumers as being applied less often than staff rated themselves as applying it. Although consumer application rating means for these items were high (*M* = 3.35 and *M* = 3.32 out of 4), staff ratings were significantly higher (*M* = 3.76 and *M* = 3.80 out of 4). This disparity may indicate a bias on the part of practitioner staff ratings, which is common to practitioner self-reports of adherence to a practice approach [54]. However the finding that consumer ratings were also high suggests that both groups viewed these elements as being frequently applied.

### Relationship between importance and frequency

For the third question, perceptions of importance of practice elements of the CRM were related to the frequency that consumers and staff practitioners report such practices are engaged in during support sessions. This finding is congruent with existing research that has found that positive attitudes about the value of an evidence base practice not only increase the likelihood that a consumer will engage in the approach, but also that practitioners will be more likely to implement an approach [44–46].

### Relationship between importance, frequency, and helpfulness of sessions

Though the primary goal of this component of study was to assess the implementation and value of key practice elements of the model, we found meaningful variability when ratings of overall ‘helpfulness of sessions to assist recovery’ were added to the analysis. Initial analysis revealed that the majority (93%) of consumers rated the sessions with staff practitioners as being helpful or very helpful towards assisting recovery. Staff perceptions about helpfulness of sessions were somewhat lower with 76% rating sessions at the helpful or very helpful level and 24% at the moderately helpful level.

Correlations revealed that perceptions of helpfulness of sessions were related to the frequency that the key practice elements were applied, both at the individual item level and the total scale level. Higher ratings of CRM application were associated with higher levels of perceived helpfulness of sessions to assist recovery. The implementation of ‘encouragement to set tasks to complete between support visits to achieve own goals’ demonstrated the strongest association (*r* = .40) with consumer perceptions of helpfulness of sessions to assist recovery. This finding stands in contrast to other findings from this study where task setting was perceived as least important and the least frequently implemented element.

Encouragement to set tasks refers to a process whereby staff and consumers collaborate to break down a goal into individual actions and strategies for a consumer to complete between support contacts. The actions and their associated goals are then reviewed at follow-up engagement sessions. The process is informed by research on health behaviour change and therapeutic homework; and functions to improve self-management, self-monitoring and self-efficacy [48]. Task setting activities in this study were rated as least important but the correlations between how frequently this activity was engaged in had the highest correlation with session helpfulness. It is unlikely that respondents were aware of the relationship between task setting frequency and perceptions of helpfulness. Had they been, it is possible that respondents might have rated its importance higher. Regardless of perceptions of value, the strength of association found in this study adds to findings in previous studies that task setting is positively related to improved outcomes [61]. There is need for services to highlight the importance of these action planning components and to increase attention to implementation of this component.

The pattern of relations shows that consumer perceptions of helpfulness of sessions were more strongly associated with application of the key CRM practice elements than staff perceptions of helpfulness with application. The disparity raises questions about whether staff may underestimate the value and impact of the work they are doing. The greatest disparity was for ‘help with motivation’ (consumers *r *= .32, staff *r* = .12). The value of ‘non-technical’ aspects of practice, such as the therapeutic relationship, meaning making and the mobilisation of hope are often underestimated by mental health practitioners [18, 25]. Although the CRM frames a collaborative engagement style and the establishment of a positive therapeutic alliance as being central to a service practice, staff may not link the experience of receiving support framed in this manner to ‘help with motivation’. For people accessing mental health services, the connection, support and safety of a trusting relationship with a practitioner who is personally invested in them and demonstrates respectful curiosity can act as a stepping stone for recovery [25]. In this study, consumer reports indicate that staff appear to deliver components of CRM that help with motivation at a high level, yet staff may underestimate the source and value of this experience to people who use the service.

### Strengths and limitations

The focus of this study is important in terms of advancing knowledge and evidence for the translation of recovery and wellbeing oriented approaches into mental health service practice. The use of matched measures to examine consumer and staff perspectives provide comparison points to substantiate findings. A clear limitation of this study is that it utilised a measure with relatively limited psychometric testing. Whilst preliminary reliability and face validity has been found, further testing is required [45] and therefore results should be interpreted with caution. Furthermore, the measure used in the current study was designed to balance existing validity with what was reasonably practical to use in a mental health setting. The measure may not have captured the full extent or the complexity of CRM principles and practices. This may limit the effectiveness of the measure to provide a comprehensive evaluation of implementation and value [54]. A common limitation of self-report measures is the potential for bias. For this study, the use of matched measures and the involvement of consumer researchers was included to minimise bias.

In total 116 consumers and 62 staff participated in this study. However, sample sizes ranged from 106 to 111 due to missing data and represent a percentage loss of between 4.3 and 8.6%. Three consumer participants did not have ‘matched data’ due to one staff member declining to participate and two being unavailable to participate having left employment at the study setting. Additional missing data may be the result of data entry error or items not responded to by participants. As such, this may have had an effect on the results.

The study was descriptive and correlational so causal explanations cannot be made. Implementation literature has demonstrated that a complex variety of factors, at different levels, interact and contribute to whether or a not mental health practice approach is translated into service delivery practice [32]. Given the extensive evidence for translational challenges the developers and researchers sought to firstly assess the feasibility of implementation within one setting. This study provides an indication that the key practice elements of the model were detected as being implemented by consumers and staff. There is a need to extend this enquiry by exploring whether or not the same results would be achieved in other settings. In particular, it is important to know whether settings that are more traditionally clinically oriented are able to obtain similar results. Furthermore, without a comparison cohort it is impossible to determine whether the results would be any different to that in any other service setting context where the CRM has not been implemented. However, prior research using equivalent measures have demonstrated differences in perceptions of those receiving CRM framed support and those not receiving CRM [45]. What the current study adds, are staff perspectives to further verify these findings and extended this to an organisation that has been implementing CRM and associated coaching programs over 5+ years.

The response rate (44%) for consumer participation in this study was relatively low and may impact on the reliability of the results. There is a chance that consumers who chose to participate were those more satisfied with the service. However steps were taken in the recruitment procedure to limit such bias, which may also have impacted the response rates. Firstly, recruitment for this study was undertaken by consumer researchers without any previous relationship with the consumers contacted for potential participation. Recruitment rates may have been higher if consumers had been recruited by staff practitioners with whom they had existing relationships. However, in order to minimise perceptions of coercion and limit bias the study involved consumer researchers to invite participants. Secondly, recruitment to participate in mental health research is often influenced by clinician decision making as to whether a person is deemed suitable (in terms of situational and mental health readiness). In this study we sought to limit clinician decision making and optimise consumer choice for opting in or out of participation [62]. Given the time and commitment involved in participation there is the potential that consumers with more obstacles may have chosen not to participate. By offering a financial incentive at recruitment, consumer participants may have been more willing to overcome obstacles to participate and/or may have been more willing to believe that their participation would be of value.

### Implications for mental health services field

The findings presented in this paper suggest that recovery and wellbeing oriented knowledge and evidence, are translatable to real world mental health service settings. Since the model’s inception, the researchers/developers worked closely with real world mental health service settings and consumers to develop and refine the model for use in everyday practice [7, 23, 63]. This involved development and testing of methods and processes to improve practitioner uptake and sustainability of practice over time [31, 39, 42, 45, 63, 64]. Complementing this work, at the service practice level; organisational readiness for change, leadership practices, and adoption of the model as a ‘whole of organisation; systems, policies and practice approach’, may have contributed to the perceptions of high implementation reflected in the results of this study.

## Conclusions

Translating recovery-oriented knowledge into mental health service systems and practice has proven to be a persistent challenge [32]. Assessing implementation can provide critical knowledge to address research to practice gaps and inform translation practices [65]. This paper presents a unit of analysis within a wider study aimed at assessing fidelity to the Collaborative Recovery Model within an Australian community managed mental health organisation. Perceptions of consumers and staff were examined regarding the value and implementation of key practice elements that are considered to reflect the model’s theoretical underpinnings and therapeutic structure.

Findings revealed that the key practice elements were perceived as being applied during service interactions at a high level and perceived by consumers and staff as being valuable for assisting recovery. There was a substantial level of agreement between consumer and staff perceptions of value and application frequency indicating visibility and positive engagement with the CRM approach in the study context. Congruent with existing research, higher perceptions of value were found to be associated with higher levels of implementation, strengthening the proposition that positive attitudes about the value of an approach can increase the likelihood of consumer engagement and staff uptake [44–46]. Variability between consumer and staff perceptions found at the individual item level highlighted areas for attention that can impact on the helpfulness of sessions to assist with recovery.

By combining recovery knowledge and policy with practices known to enhance mental health and wellbeing, along with a coaching orientation and organisational change principles, the CRM framework may offer a practical solution for mental health services to translate recovery policy into practice [48]. Further attention to the complementary nature of recovery knowledge and positive psychology and wellbeing approaches may serve to advance a whole of systems orientation to practices shown to enhance mental health and wellbeing.

## Additional files

**Additional file 1.** CEO-CRM Consumer Evaluation of CRM.

**Additional file 2.** SEO-CRM Staff Evaluation of CRM.

## Abbreviations

CRM: Collaborative Recovery Model
CEO-CRM: Consumer Evaluation of CRM
SEO-CRM: Staff Evaluation of CRM
ID: identification

## References

[1] Piat M, Sabetti J (2009). The development of a recovery-oriented mental health system in Canada: what the experience of commonwealth countries tells us. Can J Community Ment Health.

[2] Slade M, Amering M, Oades L (2008). Recovery: an international perspective. Epidemiol Psichiatr Soc.

[3] Amering M, Schmolke M. Recovery in mental health: reshaping scientific and clinical responsibilities. Herrman H, editor. Hoboken: Wiley-Blackwell; 2009.

[4] Andresen R, Oades L, Caputi P (2003). The experience of recovery from schizophrenia: towards an empirically validated stage model. Aust N Z J Psychiatry.

[5] Le Boutillier C, Leamy M, Bird VJ, Davidson L, Willams J, Slade M (2011). What does recovery mean in practice? A qualitative analysis of international recovery-oriented practice guidance. Psychiatr Serv.

[6] Farkas M, Gagne C, Anthony W, Chamberlin J (2005). Implementing recovery oriented evidence based programs: identifying the critical dimensions. Community Ment Health J.

[7] Oades LG, Deane FP, Crowe TP, Lambert WG, Kavanagh D, Lloyd C (2005). Collaborative recovery: an integrative model for working with individuals who experience chronic and recurring mental illness. Aust Psychiatry.

[8] Slade M, Amering M, Farkas M, Hamilton B, O’Hagan M, Panther G (2014). Uses and abuses of recovery: implementing recovery-oriented practices in mental health systems. World Psychiatry.

[9] Shepherd G, Boardman J, Slade M. Making recovery a reality. Sainsbury Centre for Mental Health. 2007. https://www.centreformentalhealth.org.uk/sites/default/files/2018-09/Making%20recovery%20a%20reality%20policy%20paper.pdf. Accessed 8 Aug 2017.

[10] Oades L, Mossman L, Slade M, Oades LG, Jarden A (2017). The science of wellbeing and positive recovery. Wellbeing, recovery and mental health.

[11] Piat M, Sofouli E, Sabetti J, Lambrou A, Chodos H, Briand C (2017). Protocol for mixed studies systematic review on the implementation of the recovery approach in mental health services. BMJ Open.

[12] Morera T, Pratt D, Bucci S (2017). Staff views about psychosocial aspects of recovery in psychosis: a systematic review. Psychol Psychother Theor Res Pract.

[13] Resnick SG, Rosenheck RA (2006). Recovery and positive psychology: parallel themes and potential synergies. Psychiatr Serv.

[14] Keyes CL (2007). Promoting and protecting mental health as flourishing: a complementary strategy for improving national mental health. Am Psychol.

[15] Clark S, Oades LG, Crowe TP (2012). Recovery in mental health: a movement towards well-being and meaning in contrast to an avoidance of symptoms. Psychiatr Rehabil J.

[16] Linley PA, Joseph S, Harrington S, Wood AM (2006). Positive psychology: past, present, and (possible) future. J Posit Psychol.

[17] Slade M (2010). Mental illness and well-being: the central importance of positive psychology and recovery approaches. BMC Health Serv Res.

[18] Bracken P, Thomas P, Timimi S, Asen E, Behr G, Beuster C (2012). Psychiatry beyond the current paradigm. Br J Psychiatry.

[19] Slade M, Oades L, Jarden A, Slade M, Oades LG, Jarden A (2017). Why wellbeing and recovery?. Wellbeing, recovery and mental health.

[20] Jacob S, Munro I, Taylor BJ, Griffiths D (2017). Mental health recovery: a review of the peer-reviewed published literature. Collegian.

[21] Deegan PE (1990). Spirit breaking: when the helping professions hurt. Humanist Psychol.

[22] Mancini AD (2008). Self-determination theory: a framework for the recovery paradigm. Adv Psychiatr Treat.

[23] Oades L, Crowe T, Nguyen M (2009). Leadership coaching transforming mental health systems from the inside out: the Collaborative Recovery Model as person-centred strengths based coaching psychology. Int Coach Psychol Rev.

[24] Baumann A. Statement on the meaning of empowerment of mental health service users and carers. In: Health W-EPPoUEiM, editor. Copenhagen: WHO Regional Office for Europe; 2010.

[25] O’Keeffe D, Sheridan A, Kelly A, Doyle R, Madigan K, Lawlor E, et al. ‘Recovery’ in the real world: service user experiences of mental health service use and recommendations for change 20 years on from a first episode psychosis. Administration and policy in mental health and mental health services research. 2018. 10.1007/s10488-018-0851-4 PMC5999190 29411173

[26] Leamy M, Bird V, Boutillier C, Williams J, Slade M (2011). Conceptual framework for personal recovery in mental health: systematic review and narrative synthesis. Br J Psychiatry.

[27] Bora R, Leaning S, Moores A, Roberts G (2010). Life coaching for mental health recovery: the emerging practice of recovery coaching. Adv Psychiatr Treat.

[28] Oades L, Anderson J (2012). Recovery in Australia: marshalling strengths and living values. Int Rev Psychiatry.

[29] Fairhurst K, Weavell W (2011). Co-designing mental health services—providers, consumers and carers working together. New Paradigm.

[30] Ellison ML, Belanger LK, Niles BL, Evans LC, Bauer MS (2018). Explication and definition of mental health recovery: a systematic review. Adm Policy Ment Health.

[31] Williams VC, Deane FP, Oades LG, Crowe TP, Ciarrochi J, Andresen R (2016). Enhancing recovery orientation within mental health services: expanding the utility of values. J Ment Health Train Educ Pract.

[32] Tansella M, Thornicroft G (2009). Implementation science: understanding the translation of evidence into practice. Br J Psychiatry.

[33] Le Boutillier C, Chevalier A, Lawrence V, Leamy M, Bird V, Macpherson R (2015). Staff understanding of recovery-orientated mental health practice: a systematic review and narrative synthesis. Implement Sci.

[34] Davidson L, Roe D (2007). Recovery from versus recovery in serious mental illness: one strategy for lessening confusion plaguing recovery. J Ment Health.

[35] Shanks V, Williams J, Leamy M, Bird VJ, Le Boutillier C, Slade M (2013). Measures of personal recovery. Psychiatr Serv.

[36] Burgess P, Pirkis J, Coombes T, Rosen A (2011). Assessing the value of existing recovery measures for routine use in Australian mental health services. Aust N Z J Psychiatry.

[37] Davidson L, O’Connell MJ, Tondora J, Lawless M, Evans AC (2005). Recovery in serious mental illness: a new wine or just a new bottle?. Prof Psychol Res Pract.

[38] Bellack AS, Drapalski AMY (2012). Issues and developments on the consumer recovery construct. World Psychiatry.

[39] Deane F, Andresen R, Crowe T, Oades L, Ciarrochi J, Williams V (2013). A comparison of two coaching approaches to enhance implementation of a recovery-oriented service model. Adm Policy Ment Health.

[40] Kretlow AG, Bartholomew CC (2010). Using coaching to improve the fidelity of evidence-based practices: a review of studies. Teach Educ Spec Educ.

[41] Aarons GA, Hurlburt M, Horwitz SM (2011). Advancing a conceptual model of evidence-based practice implementation in public service sectors. Adm Policy Ment Health.

[42] Deane FP, Crowe TP, King R, Kavanagh DJ, Oades LG (2006). Challenges in implementing evidence-based practice into mental health services. Aust Health Rev.

[43] Gilburt H, Slade M, Bird VJ, Oduola S, Craig T (2013). Promoting recovery-oriented practice in mental health services: a quasi-experimental mixed-methods study. BMC Psychiatry.

[44] Prendergast ML (2011). Issues in defining and applying evidence-based practices criteria for treatment of criminal-justice involved clients. J Psychoactive Drugs.

[45] Marshall S, Oades L, Crowe T (2009). Mental health consumers’ perceptions of receiving recovery-focused services. J Eval Clin Pract.

[46] Pagoto SL, Spring B, Coups EJ, Mulvaney S, Coutu M-F, Ozakinci G (2007). Barriers and facilitators of evidence-based practice perceived by behavioral science health professionals. J Clin Psychol.

[47] Oades L, Deane F, Crowe T, Boniwell I, David S (2013). The Collaborative Recovery Model: positive organisations enabling the use of strengths and values to serve recovery in enduring mental illness. Oxford handbook of happiness.

[48] Oades LG, Deane FP, Crowe TP, Slade M, Oades L, Jarden A (2017). Collaborative Recovery Model: from mental health recovery to wellbeing. Wellbeing, recovery and mental health.

[49] Oades L, Crowe T, Deane F. Life Journey Enhancement Tools (LifeJET). Illawarra Institute for Mental Health Research Online - University of Wollongong. 2008. https://ro.uow.edu.au/cgi/viewcontent.cgi?referer=https://www.google.com.au/&httpsredir=1&article=1140&context=hbspapers. Accessed 13 Dec 2017.

[50] Crowe T, Deane F, Oades L, Caputi P, Morland K (2006). Effectiveness of a collaborative recovery training program in Australia in promoting positive views about recovery. Psychiatr Serv.

[51] Piat M, Briand C, Bates E, Labonté L (2016). Recovery communities of practice: an innovative strategy for mental health system transformation. Psychiatr Serv.

[52] Slade M, Williams J, Bird V, Leamy M, Le Boutillier C (2012). Recovery grows up. J Ment Health.

[53] Roberts G, Boardman J (2014). Becoming a recovery-oriented practitioner. Adv Psychiatr Treat.

[54] Mowbray C (2003). Fidelity criteria: development, measurement, and validation. Am J Eval.

[55] Breitenstein SM, Gross D, Garvey CA, Hill C, Fogg L, Resnick B (2010). Implementation fidelity in community-based interventions. Res Nurs Health.

[56] Department of Health and Aging (2013). National Mental Health Report 2013: tracking progress of mental health reform in Australia 1993–2011.

[57] Hancock N, Bundy A, Tamsett S, McMahon M (2012). Participation of mental health consumers in research: training addressed and reliability assessed. Aust Occup Ther J.

[58] Oades LG, Law J, Marshall SL (2011). Development of a consumer constructed scale to evaluate mental health service provision. J Eval Clin Pract.

[59] Crowe TP, Deane LG, Ciarrochi J, Williams VC (2011). Parallel processes in clinical supervision: implications for coaching mental health practitioners. Int J Evid Based Coach Ment Health Pract.

[60] Deane F, Crowe T, King R, Loyd C, Meehan T (2007). Building and maintaining a recovery focused therapeutic relationship. Handbook of psychosocial rehabilitation.

[61] Kelly PJ, Deane FP (2009). Relationship between therapeutic homework and clinical outcomes for individuals with severe mental illness. Aust N Z J Psychiatry.

[62] Pinfold V, Cotney J, Hamilton S, Weeks C, Corker E, Evans-Lacko S (2017). Improving recruitment to healthcare research studies: clinician judgements explored for opting mental health service users out of the time to change viewpoint survey. J Ment Health.

[63] Marshall S, Oades L, Crowe T (2010). Australian mental health consumers’ contributions to the evaluation and improvement of recovery-oriented service provision. Isr J Psychiatry Relat Sci.

[64] Uppal S, Oades LG, Crowe TP, Deane FP (2010). Barriers to transfer of collaborative recovery training into Australian mental health services: implications for the development of evidence-based services. J Eval Clin Pract.

[65] Breitenstein SM, Fogg L, Garvey C, Hill C, Resnick B, Gross D (2010). Measuring implementation fidelity in a community-based parenting intervention. Nurs Res.

