# Supplementary material for: Consumer and staff perspectives of the implementation frequency and value of recovery and wellbeing oriented practices
Source: Int J Ment Health Syst. 2018 Oct 20;12:60. doi: 10.1186/s13033-018-0244-9 (PMC6195683; doi:10.1186/s13033-018-0244-9)
Supplement: Supplementary file 1 — Additional file 1. CEO-CRM Consumer Evaluation of CRM. [file 13033_2018_244_MOESM1_ESM.pdf]

Consumer Evaluation of the Collaborative Recovery Model (CRM) – Part A

ID no:______________________ **Date completed:____/____/_____**

Some of the statements below refer to the 'recovery process.' Psychological recovery can be defined as a process whereby individuals acquire hope and self determination to lead a meaningful life and achieve a positive sense of self, whether or not mental illness is present (Andresen, Oades & Caputi, 2002).

Many consumers have found the following areas useful in assisting their recovery. However, Recovery varies for each person. While you may think that all of these areas are important in general, we would like to know about which areas are ***more important for you personally***.

Please read through all the statements first and then go back and tick the most appropriate answer for each item.

	Not at all important	Moderately important	Important	Very important
**1.** How important is it for you that your Neami worker encourages you to take charge of your own wellbeing and recovery.	☐	☐	☐	☐
**2.** How important is it for you that your Neami worker involves you in considering choices and decisions about your recovery.	☐	☐	☐	☐
**3.** How important is it for you that your Neami worker shows respect for your right not to have to take his/her advice.	☐	☐	☐	☐
**4.** How important is it for you that your Neami worker helps to motivate you.	☐	☐	☐	☐
**5.** How important is it for you that your Neami worker understands your range of needs.	☐	☐	☐	☐
**6.** How important is it for you that your Neami worker helps you to reflect/clarify what is important to you.	☐	☐	☐	☐
**7.** How important is it for you that your Neami worker helps you to identify your strengths.	☐	☐	☐	☐
**8.** How important is it for you that your Neami worker encourages you to set goals that are personally meaningful to you.	☐	☐	☐	☐
**9.** How important is it for you that your Neami worker encourages you to set tasks to complete between support visits to achieve your own goals.	☐	☐	☐	☐

Consumer Evaluation of the Collaborative Recovery Model (CRM) – Part B

ID no:______________________ **Date completed:____/____/_____**

Section 1

We would now like to know about your experience of receiving assistance from your Neami support worker in the same areas (as part A of this survey) that consumers identify as being useful in assisting recovery.

Please consider how often your Neami worker works with you in the following ways.

Tick the most appropriate answer for each item.

	Never	Occasionally	Sometimes	Usually	Always
**1.** My Neami worker encourages me to take charge of my own wellbeing and recovery.	☐	☐	☐	☐	☐
**2.** My Neami worker involves me in considering choices and decisions about my recovery.	☐	☐	☐	☐	☐
**3.** My Neami worker shows respect for my right not to have to take advice.	☐	☐	☐	☐	☐
**4.** My Neami worker helps motivate me.	☐	☐	☐	☐	☐
**5.** My Neami worker understands my range of needs.	☐	☐	☐	☐	☐
**6.** My Neami worker helps me to reflect/clarify what is important to me.	☐	☐	☐	☐	☐
**7.** My Neami worker helps me to identify my strengths.	☐	☐	☐	☐	☐
**8.** My Neami worker encourages me to set goals that are personally meaningful to me.	☐	☐	☐	☐	☐
**9.** My Neami worker encourages me to set tasks to complete between support visits to achieve my own goals.	☐	☐	☐	☐	☐

Section 2

Please consider generally how helpful or unhelpful the sessions with your Neami worker have been in assisting in your personal recovery process over the past three months. Tick the appropriate box.

☐	☐	☐	☐
**Not at all helpful**	**Moderately helpful**	**Helpful**	**Very helpful**
