# Supplementary material for: Consumer and staff perspectives of the implementation frequency and value of recovery and wellbeing oriented practices
Source: Int J Ment Health Syst. 2018 Oct 20;12:60. doi: 10.1186/s13033-018-0244-9 (PMC6195683; doi:10.1186/s13033-018-0244-9)
Supplement: Supplementary file 2 — Additional file 2. SEO-CRM Staff Evaluation of CRM. [file 13033_2018_244_MOESM2_ESM.pdf]

Staff Evaluation of the Collaborative Recovery Model (CRM) – Part A

Consumer ID no:______________________ **Date completed:____/____/_____**

Some of the statements below refer to the 'recovery process.' Psychological recovery can be defined as a process whereby individuals acquire hope and self determination to lead a meaningful life and achieve a positive sense of self, whether or not mental illness is present (Andresen, Oades & Caputi, 2002).

Many consumers have found the following areas useful in assisting their recovery. Recovery however varies for each person. While you may think that all of these areas are important in general, we would like to know about which areas are ***more important for this particular consumer***.

Please read through all the statements first and then go back and tick the most appropriate answer for each item.

		Not at all important	Moderately important	Important	Very important
**1.**	Encouraging this consumer to take charge of his/her own wellbeing and recovery.	☐	☐	☐	☐
**2.**	Involving this consumer in considering choices and decisions about his/her recovery.	☐	☐	☐	☐
**3.**	Respecting this consumer's right not to have to take my advice.	☐	☐	☐	☐
**4.**	Helping to motivate this consumer.	☐	☐	☐	☐
**5.**	Understanding this consumer's range of needs.	☐	☐	☐	☐
**6.**	Facilitating discussions or activities that help this consumer to reflect on and clarify what is important to him/her.	☐	☐	☐	☐
**7.**	Facilitating discussions or doing activities that help this consumer to recognise his/her strengths.	☐	☐	☐	☐
**8.**	Encouraging this consumer to set goals that are personally meaningful to him/her.	☐	☐	☐	☐
**9.**	Encouraging this consumer to set tasks to complete between support visits to achieve his/her goals.	☐	☐	☐	☐

Staff Evaluation of the Collaborative Recovery Model (CRM) – Part B

Consumer ID no:_______________________ **Date completed:____/____/_____**

Section 1

Please consider how often you worked with this consumer in the following ways over the past 3 months.

Tick the most appropriate answer for each item.

		Never	Occasionally	Sometimes	Usually	Always
**1.**	I encouraged this consumer to take charge of his/her own wellbeing and recovery.	☐	☐	☐	☐	☐
**2.**	I involved this consumer in considering choices and decisions about his/her recovery.	☐	☐	☐	☐	☐
**3.**	I showed respect for this consumer's right not to have to take my advice.	☐	☐	☐	☐	☐
**4.**	I helped this consumer with motivation.	☐	☐	☐	☐	☐
**5.**	I understand this consumer's range of needs.	☐	☐	☐	☐	☐
**6.**	I facilitated discussions or activities to help this consumer to reflect on and clarify what is important to him/her.	☐	☐	☐	☐	☐
**7.**	I facilitated discussions or activities to help this consumer recognize his/her strengths.	☐	☐	☐	☐	☐
**8.**	I encouraged this consumer to set goals that are personally meaningful to him/her.	☐	☐	☐	☐	☐
**9.**	I encouraged this consumer to set tasks to complete between support visits to achieve his/her own goals.	☐	☐	☐	☐	☐

Section 2

Please consider generally how helpful or unhelpful the sessions with this consumer have been in assisting in his/her personal recovery process over the past three months. Circle the most appropriate number on the scale.

☐	☐	☐	☐
**Not at all helpful**	**Moderately helpful**	**Helpful**	**Very helpful**
